# Evaluating Sustainability of Cropland Use in Yuanzhou County of the Loess Plateau, China Using an Emergy-Based Ecological Footprint

**DOI:** 10.1371/journal.pone.0118282

**Published:** 2015-03-04

**Authors:** Xiaomei Bai, Zhongming Wen, Shaoshan An, Bicheng Li

**Affiliations:** 1 Research Center of Soil & Water Conservation and Ecological Environment, Chinese Academy of Sciences and Ministry of Education, Yangling, Shaanxi, China; 2 Institute of Soil and Water Conservation, Northwest A&F University, Yangling, Shaanxi, China; 3 Graduate University of Chinese Academy of Sciences, Beijing, China; Oklahoma State University, UNITED STATES

## Abstract

Evaluating the sustainability of cropland use is essential for guaranteeing a secure food supply and accomplishing agriculture sustainable development. This study was conducted in the ecologically vulnerable Loess Plateau region of China to evaluate the sustainability of cropland use based on an ecological footprint model that integrates emergy analysis. One modified method proposed in 2005 is known as the emergetic ecological footprint (EEF). We enhanced the method by accounting for both the surface soil energy in the carrying capacity calculation and the net topsoil loss for human consumption in the EF calculation. This paper evaluates whether the cropland of the study area was overloaded or sustainably managed during the period from 1981 to 2009. Toward this end, the final results obtained from EEF were compared to conventional EF and previous methods. The results showed that the cropland of Yuanzhou County has not been used sustainably since 1983, and the conventional EF analysis provided similar results. In contrast, a deficit did not appear during this time period when previous calculation methods of others were used. Additionally, the ecological sustainable index (ESI) from three models indicated that the recently used cropland system is unlikely to be unsustainable.

## Introduction

Cropland serves as one of the most essential resources that provides for the existence and sustainable development of human society. Currently in China, the limited availability of cropland is critically restricting agricultural production [1]. According to statistics from the United Nations Food and Agriculture Organization (FAO), China manages about 7.9% of the total cultivated land area worldwide and is responsible for feeding greater than 1.3 billion people or about 20% of the world’s population [2]. Recent rapid population growth and economic development has further increased the pressure on China’s cropland. For instance, China has a relatively low per-capita occupancy of cropland. The average cropland area per person in China was only 0.106 ha and 0.092 ha in 1996 and 2007, respectively, which is less than 40% of the world's average [3–5]. In addition, in many regions in China, and especially in ecologically fragile areas, increased farming activity and demand for additional cropland have resulted in serious environmental problems related to poor vegetative conditions and land degradation. The loss of cropland and the decrease in productivity are both expected to directly affect the security of China’s food supply as well as sustainable development in the near future. This issue has become one of the most important problems in China and has caused considerable concern [4–9]. To reverse this negative trend, studies should be conducted related to how the economy, society and environment affect the availability of cropland. In addition, the conflict between short-term economic profits and long-term ecological sustainability needs to be addressed. Effective tools or indices for combined economic and environmental assessment can assist policymakers in making appropriate decisions related to agricultural policies [10].

The concept of an Ecological Footprint (EF) provides a framework for measuring human demands on natural resources in terms of the extent of the bioproductive area with a region that is required to sustain those human life and activities. Rees (1992) and Wackernagel (1996) first proposed the use of EF as an area-based indicator [11,12]. An EF quantifies the intensity of ecological impact at individual, regional, national and global scales. The EF of an area demonstrates the basic ecological conditions related to sustainability by allowing researchers to compare human impact to the planet's limited carrying capacity from one area to another [13]. Based on this concept, human demand can only be ecological sustainability when it lies within a region’s (or nation’s) ecological carrying capacity. This method has been widely used as a useful scientific tool, has been calculated for more than 52 countries and is used by additional organizations to test the sustainability of consumption patterns [13–16]. Because of its simplicity and didactic strength, the EF has become an attractive tool for gauging sustainability, and has received both support and criticism. For example, some commentators believe that the EF concept is too simplistic and static, when both natural and economic systems are all dynamic, so the model cannot consider adaptable as technology and social institutions change [17]. Moreover, some have pointed out that single index for sustainability can be misleading because it ignores many other important factors related to sustainability [12,13]. However, as Rees (2000) stated, no tool can completely and thoroughly assess sustainability, no single method will satisfy everyone and be suitable for all circumstances, which also leaves room for and the possibility of improvement [17]. Therefore, some efforts have been made to improve and develop the EF method that are designed to make it more robust and accurate, such as the Dynamic Ecological Footprint model, the EF-NPP (net primary productivity), EEF (emergetic EF) and input-output EF methods. For EF, a combination with other methods might be the best option for improvement, because it helps researchers to address specific problems and to deal with some inconsistencies of the conventional EF [18,19]. The integrated approach used in this paper to make regional assessments is emergy-based EF (another way to define EEF) which enhanced EF calculations while using emergy analysis.

Emergy analysis (EA) is another important approach that provides sustainability indicators for analysis and evaluation on the relationship of society, environment and economy. In contrast to EF, emergy analysis is an environmental accounting method that examines the use of natural capital in economic and ecological processes based on a single form of energy defined as solar emergy. By using uniform unit of emergy, it is possible to evaluate resources used to directly and indirectly generate a product or service, along with the way energy flows in an ecosystem. In an emergy analysis, the energy flows of product or process in an open system are presented in the final indicators. According to Odum (1996) and Brown (1997), emergy indicators can be defined and applied to illuminate the different aspects of sustainability, known as ELR (Environmental Loading Ratio), ESI (Emergy Sustainability index) and Renewable Carrying Capacity [20,21]. The EA was considered more robust than EF, because it allows inclusion of other flows that influence sustainability such as soil loss, human-labor and wastes into accounting [22]. As a synthetic method this tool has been widely used to evaluate ecological, agricultural, industrial, economic and social systems since Odum introduced it in early 1980s [20,21,23–25].

A group of Chinese scholars, Zhao et al. (2005), explored the link between emergy and the EF methods in 2005 in an attempt for overcome the weaknesses of EF, by developing the emergy-based EF method [26]. In calculation of EEF method, the demand and supply of natural resources are translated into more understandable and quantifiable terms. This modified method was combined to possess the strong points of both ecological footprint and emergy analysis method, such as its easiness of application and its ability of including some important categories in calculation [18]. There have been some scientific studies to apply and discuss the EEF method in a variety of both temporal and spatial scales recently. Zhao et al (2005) originally applied this approach in Gansu Province in western China [26]. Later, Chen and Chen (2006, 2007) slightly modified this method, naming it the emergetic EF, and used it to investigate the resource consumption of Chinese society from 1981 to 2001[27,28]. Liu et al. (2008) applied the EEF model to a case study of a Jiangsu Province cropland in China [29]. More recently, Siche et al. (2010) discussed the advantages and disadvantages the ecological footprint (EF), emergy analysis (EA), and improved EEF approaches based on comparisons of each method, and developed a new combined method they applied to a system in Peru. For the calculations, the internal storage of natural capital for biocapacity, soil loss and water for human consumption were considered, as opposed to Zhao’s study, which only examined external natural capital [18]. Pereira and Ortega (2012) explored the methodology more deeply; their work considered the open ocean, deserts and frozen land, areas termed “areas not occupied by humans,” as a new category for biocapacity calculations in Brazil [30]. However, few studies have examined the use of cropland resources in ecological fragile areas through an EEF method. As noted above, in the face of the serious situation related to current cultivated land resources in China, testing the demand and supply of cropland is essential. In addition, cropland is considered as one of the important resources needed to provide basic materials for consumption by humans, and so it is the major component of an EF as well as being closely related to the carrying capacity of basic ecologically productive land.

The main objective of this study was to evaluate the ecological capacity of existing cropland that residents use to sustain themselves and to determine whether this demand is currently within the cropland’s carrying capacity for sustainable development. This article used three methods to evaluate the sustainable use of cropland, the conventional EF method, the EEF method of Zhao’s calculations (2005) and an EEF we strengthened for use in cropland assessment [26]. In this work, we selected a traditional agriculture county of China’s Loess Plateau as a case study area to examine whether the cultivated land was overloaded for the period of 1981–2009. This Loess Plateau is known worldwide for its characteristic severe soil erosion and for the fragile environment found there, yet it also serves as an important agricultural region in China. In the calculations used in the EEF method, we included both topsoil loss as a section of footprint and surface soil energy in the biocapacity, in contrast to the conventional EF methods and complementing other studies. Soil erosion is not usually included in the calculation of EF; as a result the effect of soil loss on sustainability is often ignored. However, in the Loess Plateau, soil erosion and nutrient loss are important causes of land degradation. Therefore, with the area of severe soil erosion in the Loess Plateau, considering this effect in the footprint calculation provides significant data. However, questions still arise when determining how to incorporate the impact of soil loss into an EF assessment.

## Materials and Methods

### Statement of Human and Animal Rights

This study did not include any experiments involving humans or animals. No rare or endangered species were impacted by the study. The study was based mostly on published data, so no human or animal rights issues were involved in this study.

### Ecological footprint method

The conventional EF method relies on two key assumptions; that is, most human consumption can be tracked and expressed as productive areas. Calculations of EF require three main steps. First, the footprint calculation translates consumption and services into six categories productive land areas: cropland, pasture, forest, watershed, developed land and fossil energy land. Next, the biocapacity (BC) is identified by estimating the available area of biologically productive land that is required to continuously support the resources needed by a defined population and resources needed to absorb that populations waste product’s, using existing behavior and technologies. Then, sustainability is determined. When presented as the same unit, the EF and BC can be compared directly. If the footprint is larger than the supply of productive land, the difference is known as an “ecological deficit” or a “sustainability gap” [31]. Conversely, if the supply of productive area exceeds the footprint, it indicates an ecological surplus and that the ecological-economic system is sustainable.

### Emergy analysis

Emergy analysis (EA) has been used to evaluate the energy flow among complex systems and is based on the principles of energetics, systems theory and system’s ecology [32,33]. Because different types of energy do not contribute equally in an EA, to make the comparison easier, the term “emergy” is used as the amount of one type of energy directly or indirectly used to produce another form of energy, including products and services. All of the way that input energy will flow in the system are measured using a common unit of solar emergy, expressed in solar emjoules (sej), and the value of emergy can be obtained using the following equation: Emergy = available energy of an item × transformity. Transformity is defined as the emergy per unit energy (sej/J).

### Emergy-based EF

Zhao et al (2005) originally proposed and applied the modified EF model based on emergy synthesis [26]. Similar to the conventional EF, consumption and supply are divided into six categories of productive land as noted above. In contrast, the quantities of consumption and supply are expressed in units of emergy (sej).

**Biocapacity calculation**. Carrying capacity indicates the ability of a local ecosystem to provide the necessary resources for both maintaining the existence of people and achieving their sustainability. The calculation of capacity in this study considers only natural renewable resources because renewable resources can support continuous human demands while non-renewable resources become depleted over the long term [25]. Biocapacity based on emergy (EBC) can be obtained through Equation (1):

EBC=e/ED (1)

where *EBC* is the per capita carrying capacity of an area based on emergy (ha /person·yr) and *e* is the amount of emergy of the six local renewable resources per capita (sej), specifically, solar radiation energy, rain chemical potential energy, rain geopotential energy, wind kinetic energy, earth cycle energy and topsoil energy. To avoid double-counting, only the largest input of the top five items is considered in the calculations. In addition, 3% of the topsoil energy of the earth’s ecosphere is defined as a renewable resource [34]. The empower density (ED, sej/ha) is the ratio of total emergy used in the economy of a region or nation to the total area of the region or nation. Of the ED, the local emergy density (LED) is defined as the amount of emergy per unit area of a region and the global emergy density (GED) is defined as the amount of emergy per unit area of the earth (3.10×10^14^ sej/ha·yr from Folio1 [35]; The *LED* and *GED* are obtained from Equations (2) and (3), respectively:

$$LED=\frac{total\;emergy\;of\;a\;region}{area\;of\;a\;region}$$ (2)

$$\begin{array}{l} GED=\frac{total\;emergy\;of\;the\;earth}{area\;of\;the\;earth}\\ =\frac{1.583\times 10^{25}\;sej}{5.1\times 10^{14}\;m^{2}yr}\\ =3.10\times 10^{14}\;sej/ha\cdot yr \end{array}$$ (3)

The total emergy of the earth (1.583×10^25^ sej) is the sum of earth’s renewable emergy, including solar insolation, deep earth heat and tidal energy [34]. Different results are possible using LED and GED. Zhao et al. (2005) used LED to calculate footprints and GED to calculate carrying biocapacity. In the present paper, GED is used for both indicators to obtain indicators with the same unit, global hectares, to allow a convenient comparison of the results.

**Footprint calculation**. The footprint represents the magnitude of human influence on the natural environment. In this paper, we used biological consumption per capita to estimate the footprint of cropland because data for the total import and export trade volume for a county are difficult to obtain. In the calculations, we included net topsoil loss into the accounting of consumption by humans, an approach that allows the load of the cropland to be represented more accurately. When calculating the footprint, the amount of available energy from consumption items and topsoil loss were first converted into emergy via solar transformity (sej/J). Next, the per capita emergy was calculated by dividing each emergy flow by the population of the study area (sej/person). The per capita emergy was then divided by the GED or LED to obtain the footprint per capita (ha). Finally, the sum of each of the categories obtained represents the total footprint. The footprint is calculated with Equation (4):

EEF=EM/ED (4)

where *EEF* is the footprint per capita based on the emergy analysis (ha/person), and *EM* is the emergy flow of consumption items (sej), including the two categories in this study: biological resources and net topsoil loss. These two terms can be obtained using the equation EM = energy × transformity.

### Study area

**Fig 1:**
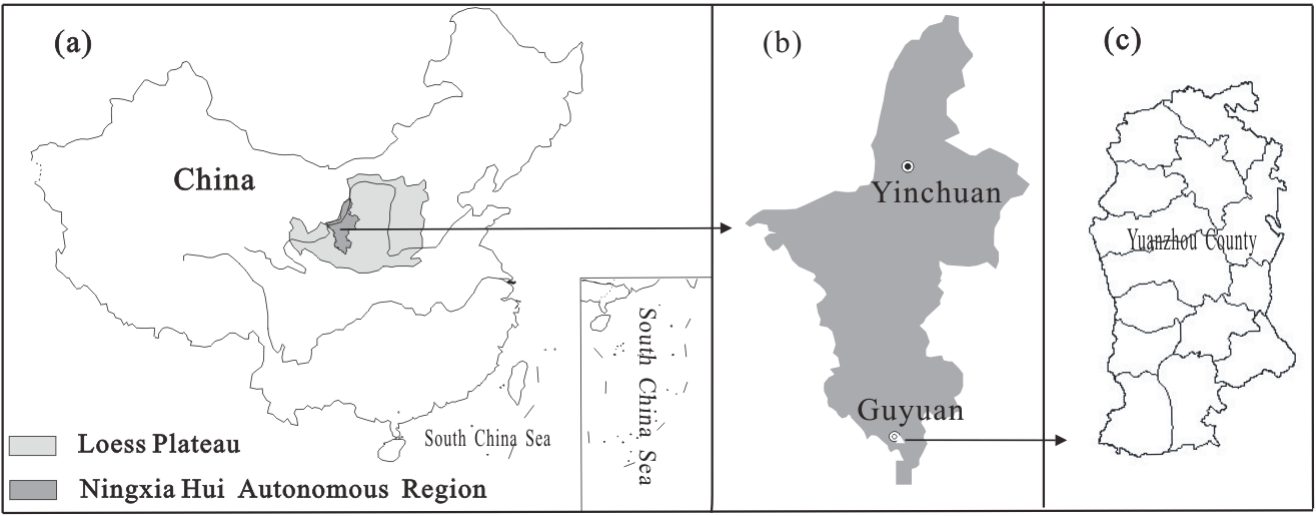
Site maps: (a) the location of Ningxia Hui Autonomous Region and the Loess Plateau, China; (b) the location of Yuanzhou County within Ningxia Hui Autonomous Region; (c) outlines of the administrative divisions of Yuanzhou County (2006).

The study area, located in Yuanzhou County (35°50′–36°20′N, 106°00′–106°30′E) in southern Ningxia Hui Autonomous Region, northwest China, encompasses of 2,756 km^2^ of land (Fig. 1). This county lies within the hill and gully region of the western Loess Plateau, with an elevation ranging from 1450 to 2500 m. The semi-arid continental temperate climate has an average annual temperature of 6.8°C with significant seasonal variation. Annual rainfall and evaporation were 428 and 1500 mm (1981–2009), respectively, a result of the region’s typically dry weather. Most precipitation falls from June to September in the form of high intensity rainstorms, which account for about 62% of the annual total and can cause severe soil erosion. About 75% of the land in this region (2072 km^2^) is eroded, and the average annual soil erosion modulus was estimated as 6000 T/km^2^ [36]. The Chinese government has taken a series of measures designed to control soil erosion and improve environmental conditions, such as terracing sloping land, prohibiting grazing in some areas and converting cropland to forest or grassland as part of the Grain for Green Program [37]. Over the past several decades, human activity has remarkably changed the quantity and ecological condition of local arable land [38–41]. Yuanzhou County, a typically semi-arid agricultural region of northwestern China, had a population of 440,568 and 104,160 ha cropland at the end of 2009; 74% of the inhabitants were farmers and sloping land comprising 78% of the farmland (slope >5°). Most of economic income of the locals comes from crop production and livestock husbandry (data sources listed in the *Data preparation* section below).

### Data preparation

Most of the original data used for this study were taken from the county statistical yearbooks (1949–2009) of Yuanzhou provided by the local government and subordinate ministries, including primarily meteorological, demographic, and crop yield data, as well as data related to the number of farms in the region. Some data, such as soil erosion data, were taken from previous research [36], and some consumption data of recent years were the results of surveys of farmers in July 2010. Large-scale land transformation occurred in the early 1980s following the economic reforms at the end of the 1970s in China. In 1999, Chinese government launched a project designed to convert cropland into woodland or grassland in ecologically fragile areas (the Grain for Green Program). Cropland in the region has gone through a process from excess reclamation to retirement since the early 1980s. It will be valuable to examine the change of footprint and capacity of cropland since 1981, which could further reflect the impacts of social and economical forces on cropland. In addition, accurate local statistics are available starting in 1981.

With 1981 selected as a viable starting point, we then used Zhao’s and conventional EF methods to calculate the footprint and biocapacity of Yuanzhou’s cropland from 1981 to 2009 allowing a comparison between the final indicators. In this study, we used the same criteria used in the traditional EF approach, i.e., ecological balance (EB) and the ESI, to conduct an ecological sustainability assessment. EB is obtained using the formula EB = BC–EF; the result indicates whether a system is in ecological deficit or surplus. ESI is calculated using the formula ESI = BC/EF. The ESI indicates whether a system can support its population based on its current lifestyle. Values lower than one indicate that the system is unsustainable; values higher than one indicate sustainability, with one as the critical turning point of sustainability [31].

## Results

### Indicators of 2009 from different methods

To demonstrate the modified approach, we present the procedure used the calculated carrying capacity and footprint from the study area in the year 2009 (Tables 1 and 2). Table 1 displays the calculation for the biocapacity of cropland using the EEF method. The calculation considered only renewable emergetic flows. The final result was obtained from the maximum emergy of external flows, such as the sun, rain, wind and earth cycle, added to topsoil (30 cm depth) emergy and was calculated at 3.2843 ha/person. The topsoil energy was included in the estimates of the local renewable biocapacity in this paper. The contribution of topsoil energy is 2.7991 ha/person, corresponding to 85% of the total carrying capacity (Table 1). This result indicates that soil organic matter contains a considerable amount of potential energy.

**Table 1:** Calculation of 2009 biocapacity for Yuanzhou County, Ningxia Hui Autonomous Region, China, using the EEF approach.

Item	Energy (J)	Transformity^a^	Total emergy^b^	Emergy per capita^c^	Biocapacity^d^
		(sej/J)	(sej)	(sej/person)	(ha/person)
Solar radiation	4.32×10^18^	1	4.32×10^18^	9.80×10^12^	0.0316
Rain, chemical	2.18×10^15^	30500	6.63×10^19^	1.51×10^14^	0.4852
Rain, geopotential	7.65×10^14^	47000	3.60×10^19^	8.16×10^13^	0.2630
Wind, kinetic energy	7.90×10^14^	2450	1.94×10^18^	4.40×10^12^	0.0142
Earth cycle	1.04×10^15^	58000	6.01×10^19^	1.36×10^14^	0.4396
Soil energy^e^	3.09×10^15^	124000	3.83×10^20^	8.69×10^14^	2.7991
Carrying capacity					3.2843

a Transformities are taken from Odum et al (2000) [35].

b Total emergy = energy × transformity.

c Population in 2009: 440,568 inhabitants.

d Biocapacity = Emergy per capita/GED (3.1×10^14^ sej/ha·yr).

e Soil energy is 3% topsoil (30 cm) energy.

**Table 2:** Calculation of 2009 footprint for Yuanzhou County, Ningxia Hui Autonomous Region, China using the EEF approach.

Item	Energy (J)	Transformity^a^	Total emergy^b^	Emergy per capita^c^	Footprint^d^
		(sej/J)	(sej)	(sej/person)	(ha/person)
**Crop production**			4.61×10^20^	1.05×10^15^	3.3732
1 Cereals	3.02×10^15^	68000	2.06×10^20^	4.67×10^14^	1.5034
2 Oilseeds	3.25×10^14^	690000	2.24×10^20^	5.09×10^14^	1.6409
3 Vegetables	2.19×10^14^	27000	5.91×10^18^	1.34×10^13^	0.0432
4 Fruits	4.79×10^13^	530000	2.54×10^19^	5.76×10^13^	0.1857
**Soil loss**	1.14×10^15^	124000	1.41×10^20^	3.20×10^14^	1.0299
**Total**			6.02×10^20^	1.37×10^15^	4.4031

a Transformities are taken or modified from Odum and Peterson (1996) [42].

b Total emergy = energy × transformity.

c Population in 2009: 440,568 inhabitants.

d Footprint = Emergy per capita/GED (3.1×10^14^ sej/ha·yr).

Table 2 shows the results of the footprint calculation for the cropland in Yuanzhou County. The calculation consists of two parts: cropland production and soil loss. The total cropland footprint obtained was 4.4031 ha per capita in 2009. Table 2 lists categories of crops that were selected after considering the actual local crop consumption and planting practices, categorized as cereals, oilseeds, vegetables and fruits. The footprint of crop production was 3.3732 ha/person, corresponding to 76.6% of the total footprint. Note that the soil loss footprint here was 1.0299 ha/person, equivalent to 23% of the total footprint. After analyzing the results and comparing the capacity and the footprint, this system of cropland was found to be in an ecological deficit.

We also obtained the final indicators of the EF using Zhao’s and the conventional EF methods for 2009 (Table 3). We obtained similar results using emergetic and conventional approaches. The capacities (3.28 and 0.31 ha/person, respectively) and the footprints (4.40 and 0.37 ha/person, respectively) for these two methods were comparable. Both methods showed that the study area has a negative ecological balance of -1.12 and -0.07 ha/person, respectively, indicating an overloaded capacity or ecological deficits. This result differed from another method of Zhao’s that calculated an ecological surplus with a positive ecological balance of 0.48 ha/person. The ecological sustainability index (ESI) calculated using EEF was 0.75, indicating that in 2009, the area was not able to support its population with its current lifestyle. This result supports the conclusions obtained with the conventional methods (0.82). Zhao’s method, however, produced an opposite result of 1.22, indicating that the area has the capacity to support 1.22 times its population given its current lifestyle.

**Table 3:** Comparison of final 2009 indicators obtained from the different ecological footprint approaches.

Indicator	EEF(ha/person)	EEF as Zhao's calculation	Conventional EF
		(ha/person)	(ha/person)
Biocapacity	3.28	2.61	0.31
Footprint	4.40	2.14	0.37
Ecological balance^a^	-1.12	0.48	-0.07
Ecological sustainability index^b^	0.75	1.22	0.82

a Ecological balance = biocapacity—footprint.

b Ecological sustainability index = biocapacity/footprint.

### Indicators changes from 1981 to 2009 using different methods

**Fig 2:**
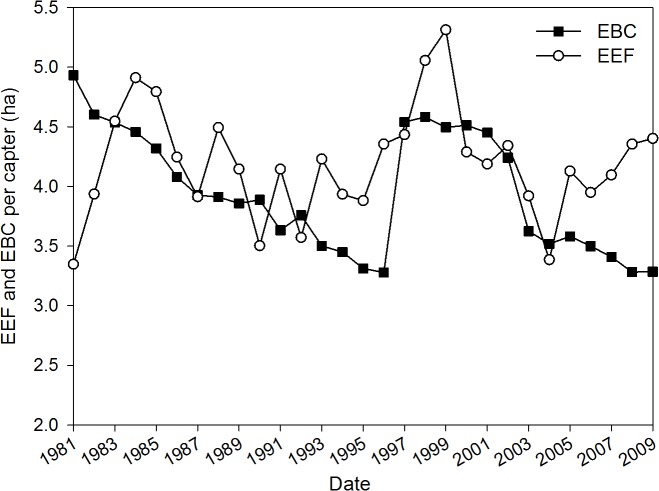
The emergetic ecological footprint (EEF) and emergetic biocapacity (EBC) per capita of cropland in Yuanzhou County, Ningxia Hui Autonomous Region, China during the period 1981–2009.

Fig. 2 presents a comparison of the study area’s footprint and its biocapacity from 1981 to 2009 as outlined above. This figure shows the emergetic footprint per capita data plotted against the emergetic capacity per capita. The curve of footprint per capita more frequently exhibits dramatic changes; its values shifted from 3.35 to 5.31 ha/person and peaked in 1999. Overall, however, the curve increases during this period. The frequent changes may be the result of instability of local agricultural productivity. In poor regions, grain accounts for most of the food consumed. Residents rely primarily on their own crop production, and the crop yield depends on the weather. The capacity curve has a flat, declining trend (4.93−3.28 ha/person) initially, although it shows a sharp and sudden increase in 1997 (3.28 to 4.54 ha/person) and then declines continuously, with a significant drop in 2003. This declining trend was primarily caused by the growth in population and was accelerated by a policy starting in 1999 that returned farmland to forest and grassland, resulting in a decrease of arable land. An increase in the total arable land in 1997 suddenly (from 118,434 to 168,734 ha) led to the sharp increase of capacity. In 2002, some of Yuanzhou’s villages and towns were reallocated into another county as a result of an adjustment in administrative districts. Consequently, 24% of the cropland was lost. A population change of 6% led the significant and distinct decrease in capacity that occurred in 2003. The differences between the curves for EF and capacity reveal the per capita ecological deficits that existed after 1983, so that an ecological surplus only appeared in some years (Fig. 2).

**Fig 3:**
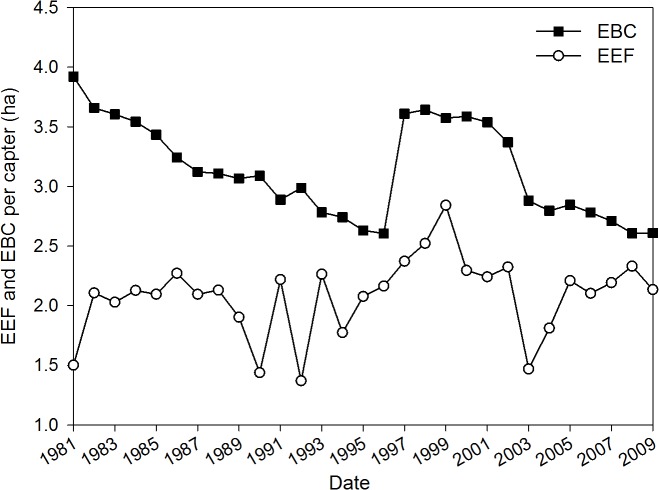
The emergetic ecological footprint (EEF) and emergetic biocapacity (EBC) per capita of cropland in Yuanzhou County, Ningxia Hui Autonomous Region, China during the period 1981–2009 as determined by Zhao’s calculation.

**Fig 4:**
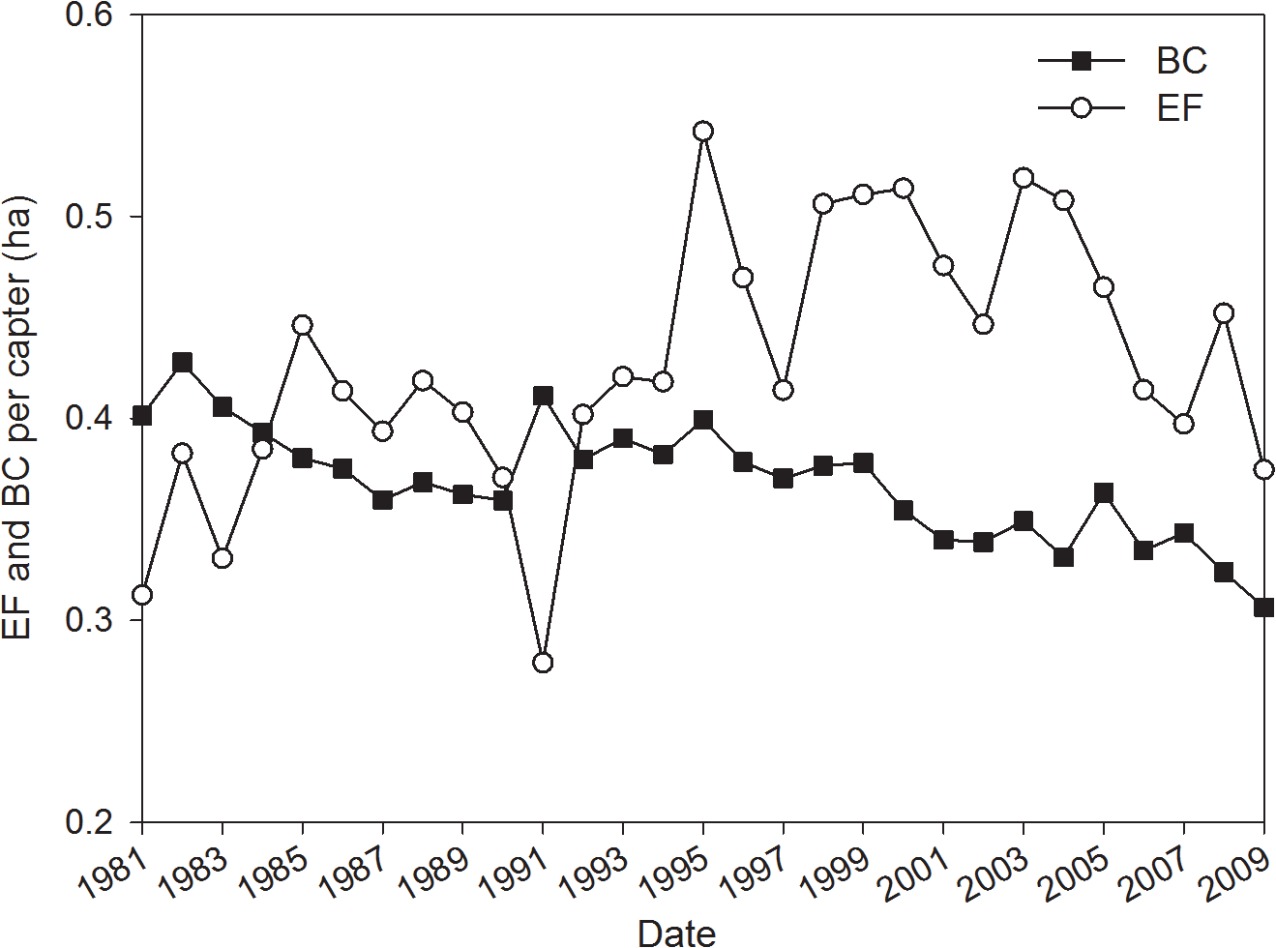
The conventional ecological footprint (EF) and biocapacity (BC) per capita for cropland in Yuanzhou County, Ningxia Hui Autonomous Region, China during the period 1981–2009.

To examine the above results in more detail, we also show results from two other approaches (Figs. 3 and 4). First, capacity and footprint per capita were shown using Zhao’s method (Fig. 3). The curves display similar trends to those from the EEF method; the footprint increased and the overall capacity decreased. In contrast, biocapacity always exceeded the county’s footprint during the time period shown here (Fig. 3), which indicates an ecological surplus. These data may be the result of using GED for the capacity and LED for the footprint. Using GED will obtain the result in global hectares. LED corresponds to results obtained in regional hectares. Remarkably, the LED (6.40×10^14^ sej/ha in 2009) is larger than the GED (3.10×10^14^sej/ha), causing the footprint to be lower than normal.

Fig. 4 shows the results from the EF method for the given period and the curves show the same trend; that is, the footprint increased and the biocapacity decreased. An ecological deficit appeared for the first time in 1985; this method shows that the deficit emerged two years later compared with the results from the EEF approach (Fig. 2). The trends shown by the per capita footprint and biocapacity are not only related to the structures of consumption and population but also to the global average crop yield in the given years, considering the differences in the algorithms. The ecological deficits from the EEF approach tend to increase in contrast to the deficits calculated by conventional EF.

**Fig 5:**
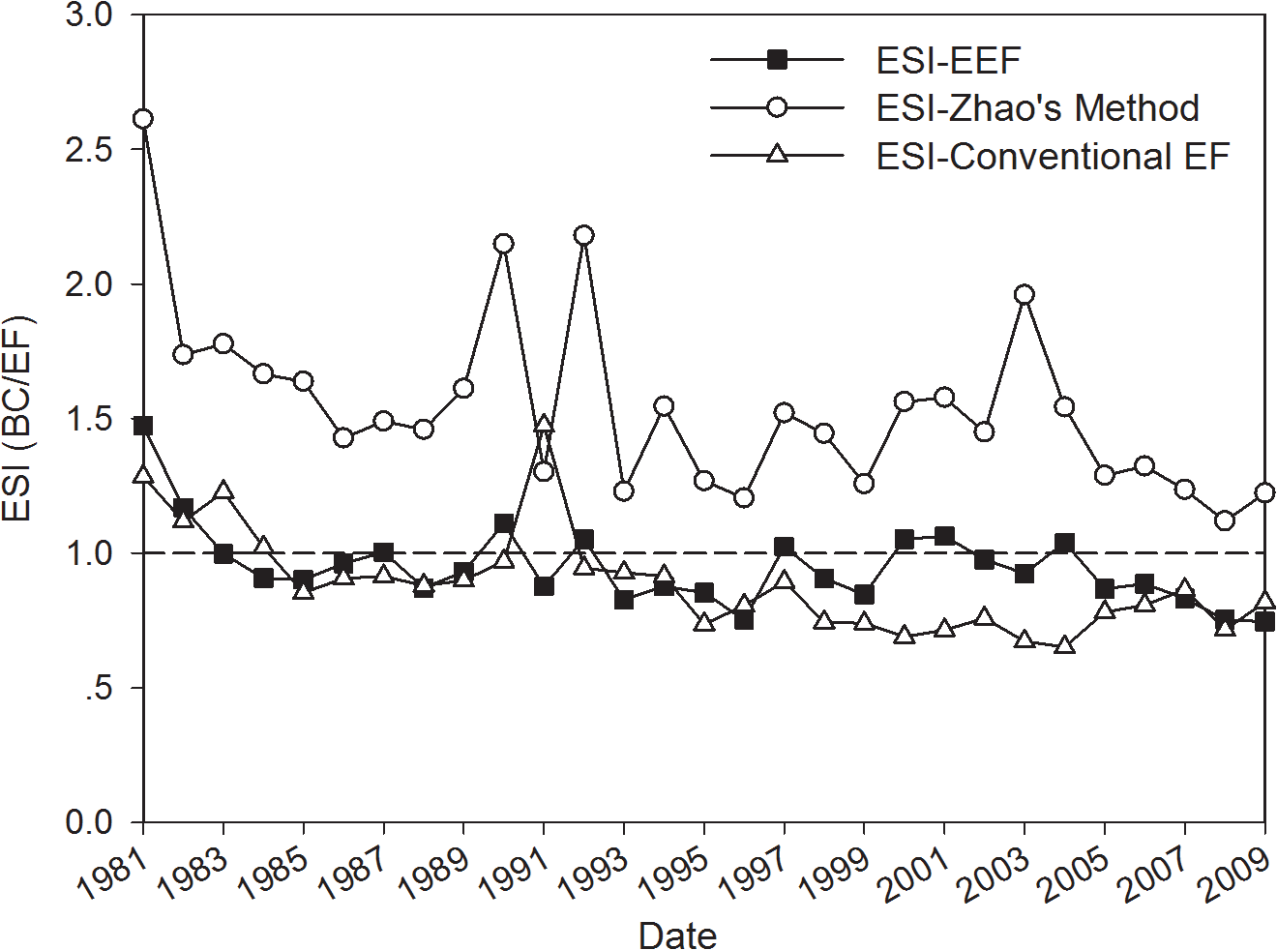
The three data series of the ecological sustainability index (ESI) during the period 1981–2009 based on three ecological footprint approaches for Yuanzhou County, Ningxia Hui Autonomous Region, China.

Fig. 5 demonstrates the ratio of the county’s footprint to the biocapacity obtained from the three different approaches outlined above. The horizontal dotted line represents the critical value of 1. The curves for ESI were lower than the line of critical limit from the results found using both the EEF and EF methods. This result indicates that the county’s cropland system became overused and the current level of use is unsustainable. The ESI as calculated by the EEF method has decreased from 1.47 to 0.75 and reached a value of less than 1 for the first time since 1984. The degree of deviation tended to increase in recent years. The ESI found using the EF method was between 1.47 and 0.71 and was lower than 1 for the first time since 1985, and its trend is approaching the limit. Based on Zhao’s method, the ESI was always above 1 but eventually decreased to nearly 1, indicating that the county could handle its population load but was approaching the critical limit, especially in recent years. Therefore, these curves illustrate the degree of overuse of the land resources, and the decline of the ESI indicates potential problems are on the horizon related to sustainable development.

## Discussion

The values of sustainable indicators were obviously different from above assessment models. The EEF method provided a negative result for the sustainability of cropland use, as did the EF model. One reason for overload of cropland is that the population has increased rapidly in this region, and another is that the amount of available cropland has decreased sharply after 1999. Because of the Grain for Green Program that is designed to convert sloping farmlands into woodlands and grasses, the footprint caused by soil erosion has been reduced, and in a short time, because land productivity has not improved significantly, the ecological capacity was also reduced due to a decrease in the spatial extent of farmland areas. However, in the long term, the Grain for Green Program can effectively help people control soil erosion, increase land productive and ensure the sustainable development of regional agriculture [38]. However, with the further implementation of that policy, the decrease in the amount of land available for agriculture will inevitably result in a reduction of farmer’s incomes. Knowing how to provide enough food for the local farmers and how to help them maintain a stable income has become a major economic problem for the local government. Consequently, the farmers have given up using the sloping cropland because they can then receive a subsidy from government; in addition, livestock breeding has recently been encouraged and supported by the government. Concurrently, reclaimed grassland and new orchard areas have increased notably, and the use of animal husbandry and the planting of orchards actually increased the income of some local farmers. Nevertheless, the effect of increasing the ecological capacity is not significant because the development project was done on a short term and at a small scale. Thus, the ecological deficit was expanding with cropland conversion. In addition, data limitations did not allow the consideration of the factor of trade in calculation of consumption; it also caused the transfer of ecological pressures among regions to be ignored, which led to an overestimate of the footprint and helped to generate an ecological deficit.

In contrast to conventional EF, the emergy-based EF method avoided influence of yield and equivalence factors that failed to consider the substantive ecological and bioregional disparities [43]. Furthermore, biocapacity was derived from a primary renewable resource, which may be more reliable for evaluating sustainability. Again, the EEF model took soil loss into account as a consumption factor in the footprint calculation; this is a more reasonable way to express the impact of this typical characteristic for the Loess Plateau on productivity and sustainability. However, the EEF has its limitations. First, it is impossible to compare biocapacity and the footprint of each item in the way the conventional approach does because biocapacity cannot be separated into categories [44]. Second, there is still a debate regarding the certainty of transformity considering the complexity of the system. However, selecting a different value for transformity will yield a significantly different result. So, these problems should be addressed in future studies. In fact, evidently, the EF allowed an evaluation only from the perspective of the economic benefit of land use, which focused on relationship between supply and demand rather than the ecological conditions of the cropland resources itself. Therefore, with the combination of EF and EA, a more well-rounded evaluation could be achieved that takes into account the effects of ecological conditions such as soil loss on sustainability; supplementing economic evaluation with ecological ones is important.

Quantifying human consumption and ecological capacity provides a common language for society to work towards sustainability. As this study shows, regional sustainable development mainly depends on the availability of local renewable resources and the wise use of those resources. Therefore, further actions are necessary for the effective conservation of natural resources, in order to improve the methods of agriculture production and the lives of local people in Yuanzhou County. Based on the results of this study, agriculture land should be managed using improved and scientifically sound methods. First, a certain quantity of cultivated land should be effectively protected to meet the basic needs of local people and to maintain the unique ecological function of each part of the landscape. Second, the farmers should be guided and supported in that government policies should be developed that support the local economy while increasing the environmental awareness of both policy makers and farmers, in a way that can ensure the basic incomes for farmers and reduce unreasonable reclamation while improving the sustainable productivity of cropland.

## Conclusions

The EF provides a measure for quantifying both the impact of human activities on the natural world and the human load that an ecosystem could tolerate. However, traditional calculation often loses its advantages when turning to the interface of economy and environment, thus underestimating or neglecting environmental impacts and inputs provided by the nature. The exploration and utilization of more systematic evaluation method is needed. In this case, the integrated use of different method could be a good alternative aiming to analyze more accurately the system’s sustainability. Hence, the combination between EF and EA was discussed and applied to cropland in this paper. The contribution presented here is the inclusion of topsoil loss in footprint and soil energy in biocapacity calculation. This category is not accounted for in EF approach, neither in Zhao’s calculation. Soil is a crucial factor influencing the sustainable use of cropland. Considering serious depletion of surface soil caused by human activities in study area, soil loss was included as consumption in the footprint calculations. Also, soil energy (3%) was considered as a renewable resource, because it provides biocapacity that sustain human being. In this way, the evaluation of sustainability seems to be more exact for measuring the anthropic impact on the nature and assessing the environmental services. We consider that which can be a valid step on improving the EEF.

In this case study of Yuanzhou County’s cropland using EEF and conventional EF, these indices of sustainability demonstrated that the cropland use in this region is not currently sustainable. (1) The EB reached -1.12 ha per capita in 2009, and this continues a trend that extended from 1981 to 2009. The conventional EF method calculated this value at -0.07 ha per capita, and this indicated that the difference of footprint and capacity was relatively small. Although the EB determined from Zhao’s calculation always had positive values over the course of this study, the values gradually decreased over time from 2.42 to 0.48 ha/person, indicating that the outlook for ecological sustainability is not optimistic. (2) The indicators of the ESI have been less than 1 since 1984 according to the EEF and conventional EF, which reveals that the county’s cropland capacity was in a state of overload and unable to satisfy the demands of the local residents given their current consumption and production patterns. Considering Zhao’s calculations, we obtained different sequential values of ESI; these values were greater than 1 during 1981–2009, but exhibited a trend that approaches 1 in recent years. This finding suggests that the sustainability of Yuanzhou’s cropland use is likely to continue to decline if no effective economic and ecological measures are implemented soon. Different indices also implied that patterns of unreasonable levels of production and consumption in this agricultural system should be changed.

Both emergetic footprint and EF express the stress on this zonal cropland. We cannot conclude that the results from enhanced EEF are more accurate than the other two, primarily because the above three methods have their limitations in calculating sustainability. Nevertheless, using a combination of methods should provide a meaningful estimate EF of cropland by adding environmental factors into the calculations in ecologically fragile areas. This will be needed to achieve a more balanced approach to the evaluation of human use of resources in the future.
